# Enhancing the Benefits of Antiretroviral Therapy in Vietnam: Towards Ending AIDS

**DOI:** 10.1007/s11904-014-0235-7

**Published:** 2014-12-04

**Authors:** Masaya Kato, Nguyen Hoang Long, Bui Duc Duong, Do Thi Nhan, Thi Thuy Van Nguyen, Nguyen Huu Hai, Le Minh Giang, Do Mai Hoa, Nguyen Thanh Van, Amitabh B. Suthar, Chris Fontaine, Patrick Nadol, Ying-Ru Lo, Michelle S. McConnell

**Affiliations:** World Health Organization Vietnam Country Office, 6 3 Tran Hung Dao Street, Hanoi, Vietnam; Vietnam Authority of HIV/AIDS Control, Ministry of Health, Hanoi, Vietnam; Hanoi Medical University, Hanoi, Vietnam; Hanoi School of Public Health, Hanoi, Vietnam; UNAIDS Vietnam, Hanoi, Vietnam; US Centers for Disease Control and Prevention, Vietnam Country Office, Hanoi, Vietnam; World Health Organization Regional Office for the Western Pacific, Manila, Philippines

**Keywords:** Antiretroviral therapy, HIV prevention, Concentrated epidemic, Vietnam, People who inject drugs

## Abstract

Vietnam has a concentrated HIV epidemic, with the highest HIV prevalence being observed among people who inject drugs (PWID). Based on its experience scaling-up robust HIV interventions, Vietnam aims to further strengthen its response by harnessing the preventive benefits of antiretroviral therapy (ART). Mathematical modelling suggests that prioritizing key populations for earlier access to ART, combined with other prevention interventions, may have significant impact on the epidemic, cost-effectively reducing new HIV infections and deaths. Pilot studies are being conducted to assess feasibility and acceptability of expansion of HIV testing and counselling (HTC) and early ART among key populations and to demonstrate innovative service delivery models to address challenges in uptake of services across the care cascade. Earlier access of key populations to combination prevention interventions, combined with sustained political commitment and supportive environment for key populations, are essential for maximum impact of ART on the HIV epidemic in Vietnam.

## Introduction

Vietnam has an HIV epidemic concentrated in key populations. In 2013, the estimated HIV prevalence in general populations (aged 15–49 years) was 0.39 % [1], while people who inject drugs (PWID) had the highest HIV prevalence at 10.3 %, followed by men who have sex with men (MSM) at 3.7 % and female sex workers (FSWs) at 2.6 %, according to the national sentinel surveillance [2]. There were an estimated 258,524 people living with HIV (PLHIV) [1] and estimated 271,000 PWID in 2013 [2]. Studies have reported PWID are the dominant populations among people receiving antiretroviral therapy [3, 4].

Vietnam has made considerable progress in implementing evidence-based combination prevention focusing on key populations. Harm reduction interventions were rapidly expanded reaching a large number of PWID [5]. At the end of 2013, 15,542 people were receiving methadone maintenance therapy (MMT) at 80 clinics [2], and in 2013, needle and syringe programmes (NSPs) distributed approximately 26.7 million needles and syringes or 98 syringes per PWID per year [2]. The condom promotion programme reached primarily FSWs and MSM distributing 14 million free condoms and additional 32 million condoms through social marketing [2, 5]. According to integrated biological and behavioural surveillance (IBBS) in 2009, among PWID surveyed in 12 provinces, a median of 94.3 % (range 85.1 to 98.0 %, by province) reported they had used new needles in the last injection. Among FSW surveyed in 10 provinces, median condom use with one-time clients at last sex was 95.2 % (range 73.8 to 99.3 %) [6]. Given the high levels of safe behaviour reported among key populations, further reduction of HIV transmission through behavioural interventions alone may not be easily achievable. According to the national technical working group on estimation and projection, an estimated 12,000 new infections will likely occur in 2014 and will continue at this level unless the current response changes [7].

Vietnam has also achieved a remarkable scale-up of antiretroviral therapy (ART); 82,687 PLHIV were receiving ART at the end of 2013, with an estimated coverage of 68 % of PLHIV in need based on current national eligibility criteria (CD4 < 350 cells/mm^3^ in adults) [2]. ART was being delivered at 364 HIV outpatient clinics at the end of 2013 [2]. With estimated 12,000 new HIV infections per year [7] and approximately 10,000 increase in the number of ART patients per year [2], Vietnam is near the programmatic ‘tipping point’ of controlling the HIV epidemic, where the annual increase in new patients on ART exceeds the annual new HIV infections [8]. With growing evidence showing strong preventive benefits of ART [9–11], Vietnam responded by exploring how further increase in access to ART could improve the national response to HIV as part of a combination prevention approach.

## Mathematical Modelling of Potential Impact of ART

Mathematical modelling can estimate the potential impact of different policy options and help determine optimal strategies to minimize HIV transmission and HIV-associated mortality. Two recent modelling studies analysed the preventive impacts of expanded ART in Vietnam, one using data from Can Tho province [12] and another using national data in a Prevtool model [13].

**Fig. 1:**
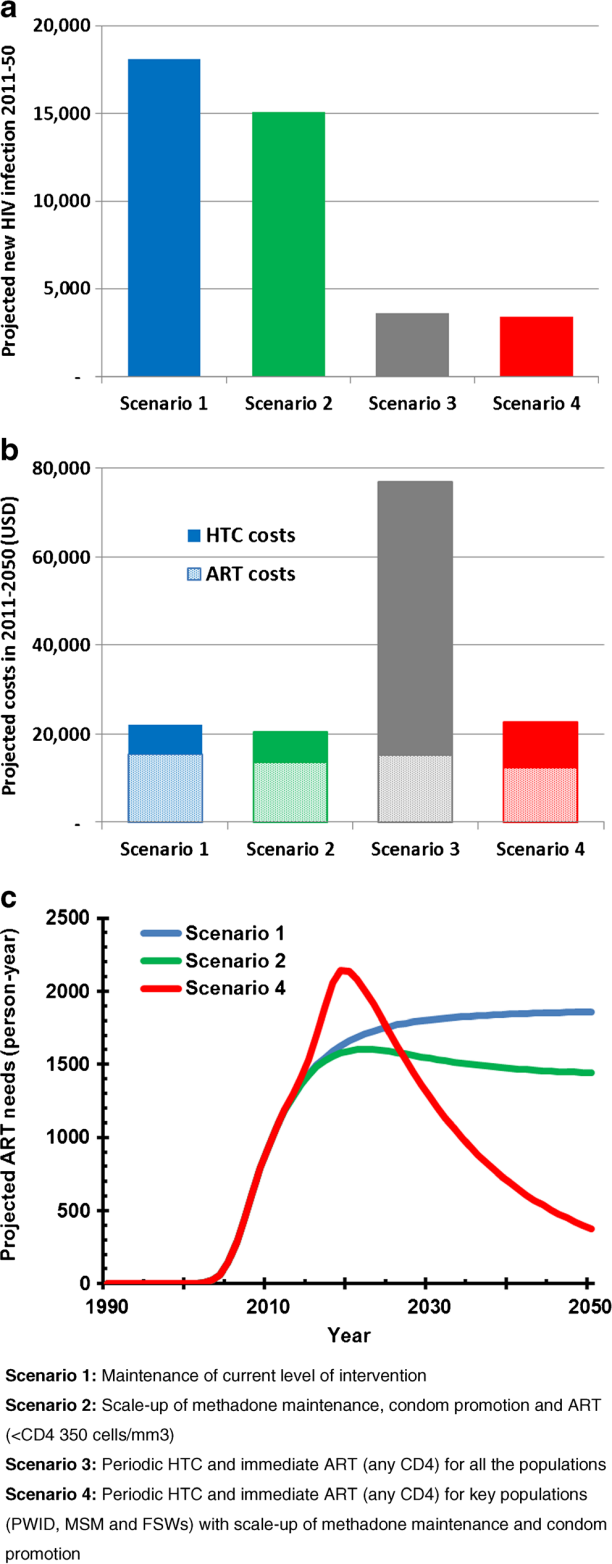
Projected cumulative new HIV infections in 2011–2050 (**a**), projected cumulative costs of HTC and ART in 2011–2050 (**b**) and time course of projected needs for ART in 2011–2050 (**c**) (adapted from [12]). In the panel **c**, scenario 3 is not presented but it shows similar pattern as scenario 4

Both models suggested early initiation of ART will avert a substantial number of new HIV infections in Vietnam [12, 13]. If ART is provided to PLHIV immediately upon diagnosis, combined with a significant expansion of HIV testing efforts to all adults resulting in earlier diagnosis, the national Prevtool model estimated a 63 % reduction of new HIV infections over a 20-year period [13], and the Can Tho model reported an 80 % reduction of new HIV infections over a 40-year period (Fig. 1a) [12]. A substantial reduction in new HIV infections should reduce the future need for ART, resulting in considerable future cost savings for the national HIV programme [12]. Expanding ART eligibility in line with WHO 2013 guidelines is also likely cost-effective. The national Prevtool model reported that the incremental cost-effectiveness ratio (per DALY averted) is $290 if the CD4 count threshold is changed from 350 cells/mm^3^ (current national guidelines) to 500 cells/mm^3^ and $289 for extending ART eligibility to all adults living with HIV [13].

While those findings described above are consistent with those from other modelling studies conducted for generalized epidemic settings, both studies also noted specific findings for Vietnam’s concentrated epidemic. That is, maximizing preventive impact of ART in a cost-effective manner requires prioritizing key populations for periodic HIV testing, followed by linkage to immediate ART; however, expanding periodic testing to the general populations in Vietnam is not cost-effective. [12, 13]. For example, the Can Tho model suggests expanding periodic testing to the general populations requires four times higher costs than focusing on key populations, primary due to the costs of testing the larger populations, to achieve similar reduction in the new infections in 2011-2050 period. (Fig. 1a, b) [12]. The Can Tho model also found that prioritizing PWID for testing and ART achieves the greatest reduction in new infections per unit investment, reflecting the situation that the highest HIV transmission occurs among PWID and from PWID to their sexual partners. This is in stark contrast to generalized epidemic settings, where general population testing campaigns may be a cost-effective option [13–15].

Injection drug use is the dominant mode of HIV transmission in Vietnam, and ART for prevention of parenteral transmission may have an impact on the overall epidemic. While studies from Vancouver [16] and Baltimore [17] reported ecological associations between community viral load and newly diagnosed HIV cases among PWID, the magnitude of preventive effects of ART on parenteral HIV transmission is unknown. However, it is plausible to presume a reduction in HIV viral load would provide some degree of protection over needle-borne HIV transmission, in addition to its proven impact on sexual transmission [18]. The Can Tho model thus examined varying the efficacy of ART for reducing needle-borne transmission from 96 to 70 %; such reduction in the efficacy attenuates the overall effectiveness, but even with the efficacy at 70 %, the model found an estimated 54 % of cumulative new infections from 2011 to 2050 could be prevented with immediate access to ART among key populations [12].

## Current Challenges and Opportunities Across the Cascade

The population-level impact of ART in preventing HIV transmission, as projected by modelling studies, is determined by the proportion of PLHIV with suppressed viral load, especially among those who bear high risk of transmitting the virus to other individuals, such as key populations, HIV-positive partners in serodiscordant relationships and pregnant women. The proportion of PLHIV with suppressed viral load is dependent on uptake and adherence to services across the cascade including testing, linkage to care and ART initiation and adherence.

### HIV Testing and Linkage to Care

Uptake of HIV testing has been limited among key populations in Vietnam. The proportion that reported receiving HIV testing and test results in the last 12 months was 24 % among male PWID, 29 % among MSM and 35 % among FSW in 2013 [2]. It is also common that people diagnosed with HIV infection are not successfully linked to care and treatment. According to the national case reporting system, 197,335 PLHIV were diagnosed, reported and presumably alive at the end of 2012, although only 72,213 people (36 %) were registered at HIV care clinics [19]. In a study conducted in Thanh Hoa province, 625 people were tested HIV positive in 2011, among whom only 382 (61 %) people were successfully linked to care [20]. This study found people are less likely to be linked from HIV testing and counselling (HTC) to care if HTC and care clinics are located at separate locations [20].

### Timely Initiation of Antiretrovirals

Late initiation of ART at advanced stage of HIV infection is associated with higher mortality and reduced retention on ART [4] and also leads to greater opportunities for HIV to be transmitted. Late presentation for care services and late initiation of HIV treatment is common in Vietnam. Nguyen et al. reported that, during 2005–2009, median CD4 count was 73 cells/mm^3^ at care enrolment and 78 cells/mm^3^ at ART initiation [4]. In more recent years, people have tended to start ART at higher CD4 counts. Among people who started ART in 2011, median CD4 count was 97 cells/mm^3^ [21]. Among the 68 clinics implementing the quality improvement programme ‘HIVQUAL’, the mean CD4 count at ART initiation was 220 cells/mm^3^ (median 197 cells/mm^3^) in 2013 [2].

Data also show that PWID, compared to those who do not inject drugs, are more likely to access ART with lower CD4 count (69 vs. 96 cells/mm^3^), and a higher prevalence of WHO stage 3 or 4 diseases (83 vs. 68 %) and active tuberculosis (16 vs. 10 %) [4]. A study at two clinics in Ho Chi Minh City showed a similar trend [3]. On the other hand, PWID receiving MMT are more likely to start ART with a higher median CD4 count, compared with PWID not receiving MMT (203 vs 80 cells/mm^3^), indicating a promising option for integrating HIV services with opioid substitution therapy [22].

### Retention in Care and Treatment

In Vietnam, once people receive ART, retention is good and similar to the rates reported from other low or middle income countries [23]. Since 2007, the annual national ART facility survey reported an average retention rate of over 80 % at 12 months, including 84.6 % among those starting ART in 2011 [2, 4, 19, 24, 25]. For the first year of ART, deaths accounted for 11.0 % of attrition, loss-to-follow-up (LTFU) for 5.6 %, and stopping ART for other reasons for 1.3 % [25]. Beyond 12 months, attrition was primarily due to LTFU, with an average retention of 78.4 % at 24 months and 74.3 % at 36 months after ART start [25]. In contrast, there are limited data on retention before starting ART (pre-ART).

### Adherence, Viral Suppression, HIV Drug Resistance

Various studies have reported adherence among people receiving ART in Vietnam. One study using audio-computer-assisted self-interview reported 25 % of people receiving ART reported having suboptimal adherence [26]. The same study also found that depression and heavy alcohol use were associated with sub-optimal adherence and that drug use alone was not associated with suboptimal adherence but interacted with heavy alcohol use to reduce adherence [26].

On the other hand, available data suggest a relatively large proportion of people on ART in Vietnam have achieved viral suppression. In a cohort study in Quanh Ninh province, which enrolled 605 patients from 2007 to 2009 and assessed viral load of over 87 % of those retained, 93.1 and 93.7 % of those tested had viral load less than 1000 copies/ml at 12 and 24 months, respectively, after ART start [27]. Among people starting ART from 2008 to 2009 in four clinics (two clinics in Ho Chi Minh City, one in Hai Phong, one in Hai Duong) and on treatment at 12 months after ART start, 94.5 % had viral load less than 1000 copies/ml and 91.3 % less than 250 copies/ml (Do TN, personal communication).

The majority of studies assessing transmitted HIV drug resistance (HIV DR) have reported that transmitted HIV DR levels in major cities in Vietnam are less than 5 % [28, 29], except one study in HCMC which reported HIV DR to nonnucleoside reverse-transcriptase inhibitors was at moderate level between 5 and 15 % [30]. The level of acquired HIV DR among people receiving ART was studied using a WHO protocol at the four clinics, and the study reported HIV DR in 2.9 % of patients at 12 months of treatment, and 12.3 % of patients were classified as having possible HIV DR (mostly lost-to-follow-up) (Do TN, personal communication).

These results support the notion that a large majority of people on ART are achieving viral control at least in the early years on treatment and that HIV DR is maintained at a low level in Vietnam. However, in order for ART to continue to be effective, sustained efforts are essential to ensure high levels of adherence and viral suppression at a population level, so as to prevent the emergence of HIV drug resistance. Understanding the potential facilitators and barriers to adherence, investing in improved support for adherence and addressing substance use and mental disorders are increasingly important.

### Stigma and Discrimination

Studies in Vietnam and other countries indicate that lack of confidentiality is a considerable barrier to efforts to expand HIV testing among key populations and accessing HIV services [31]. PLHIV and key populations in Vietnam face considerable stigma and discrimination. More than half of a representative sample of PLHIV in five provinces reported in 2011 that their right to live free of discrimination had been violated [32]. In the context of Vietnam’s concentrated epidemic, stigma is attached both to HIV status and to risky behaviours which are stigmatized in and of themselves as well as perceived to increase HIV risk—injecting drug use, sex work and homosexual activity. Many survey respondents reported their perception that their behaviours create more stigma and discrimination than their HIV status [32]. In addition, nearly 30 % of PLHIV believed that their serostatus was disclosed to others without their consent [32]. Continued efforts to address stigma, discrimination and lack of confidentiality are needed to improve uptake of and retention in services across the care cascade.

## Harnessing Preventive Effects of ART in Vietnam

### Serodiscordant Couples

Vietnam reacted quickly when the HPTN052 trial results were disseminated in 2011 [11]. Vietnam Authority of HIV/AIDS Control (VAAC) initiated planning a pilot study to assess feasibility of couples HIV testing and counselling and immediate ART for serodiscordant couples (Table 1). In Dien Bien and Can Tho provinces, 126 index cases in serodiscordant relationships who were predominantly PWID (85 % males, 58 % reporting injection drug use, 52 % CD4 > 350 cells/mm^3^) started immediate ART in 2013 [33]. Preliminary results suggest the large majority were retained (>90 %) and achieved viral suppression (78 %, <1000 copies/ml) at 3 months following ART initiation [33]. Self-reported condom use in the couples at baseline was not high (less than 80 %), but it increased during follow-up. The study also found that a large proportion of HIV-positive partners had high baseline viral load, including those with CD4 count over 350 cells/mm^3^ (eligibility threshold per current national guidelines) indicating potential risk of HIV transmission to their negative partners without ART access, in the context of low condom use. The interim review also suggested that couple HIV testing and counselling and immediate ART are feasible and potentially effective approach in Vietnam to identify serodiscordant couples and prevent transmission within the couples. While final results will be available in early 2015, these findings are accelerating discussion within the Ministry of Health regarding the adoption of early ART as a priority intervention in the national response.

**Table 1:** Initiatives to harness preventive effects of ART and to transform service delivery approaches in Vietnam

Initiatives	Provinces	Features of implementation studies and operational pilots
Immediate ART among serodiscoudant couples	Dien Bien Can Tho	Objectives: feasibility assessment of the interventions Interventions: couples HIV testing and counselling and immediate ART irrespective of CD4 count for index partners in serodiscordant couples Populations: 126 serodiscordant couples Timeframe: March 2013 to December 2014 Key outcomes: viral suppression (index partners), improved care cascade
Immediate ART among people who inject drugs	Thai Nguyen Thanh Hoa	Objectives: feasibility and acceptability assessment of the interventions Interventions: periodic voluntary HIV testing and counselling, and immediate ART irrespective of CD4 count Populations: 300 HIV positive people who inject drugs Timeframe: March 2014 to December 2015 Key outcomes: viral suppression, improved care cascade, acceptability
Integrating HIV services into primary health care (Treatment 2.0 pilot)	Dien Bien Can Tho (phase 1) Thai Nguyen Thanh Hoa (phase 2)	Objectives: feasibility assessment of service delivery model to enhance access, retention and sustainability Service delivery model: decentralization of HIV testing and treatment services into sub-district primary health care services, point-of-care HIV diagnosis and CD4 count, once-daily fixed dose combination, community mobilization Populations: key populations, their partners and pregnant women Timeframe: phase 1 from July 2012, phase 2 from April 2014 (ongoing) Key outcomes: earlier treatment initiation, improved testing uptake, timeliness of services, acceptability
Service delivery in mountainous provinces	Seven mountainous provinces	Objectives: feasibility assessment of service delivery models in remote mountainous provinces Service delivery model: decentralization of HIV testing and treatment services and immediate ART irrespective of CD4 count to address the challenges in accessing CD4 count test Populations: people residing in mountainous provinces with a focus on key populations and their partners Timeframe: from November 2014 Key outcomes: improved care cascade, earlier diagnosis, retention on ART

### Key Populations

WHO does not have recommendations for key populations to start ART earlier than other populations [34], though it discusses the potential operational and public health benefits of early ART for key populations towards decreasing HIV transmission [35]. Mathematical modelling using local data suggests that prioritizing early ART for key populations could cost-effectively and substantially reduce HIV transmission in Vietnam’s epidemic [12, 13]. Civil society advocates that PLHIV should be provided with the information on preventive effects on HIV transmission and that individuals’ autonomy should be respected in receiving HIV testing and deciding whether to start ART at higher CD4 count [36]. Careful assessment and debate, involving key populations, are essential before making a modelled strategy as part of the national response. To assess feasibility and acceptability of periodic HIV testing and immediate ART among PWID, a pilot study was developed in Thai Nguyen and Thanh Hoa provinces (Table 1). Repeated HIV testing and counselling is encouraged every 6 months by having health care workers and peer educators reach out to a greater number of PWID. If HIV positive, PWID are counselled on potential benefits and risks of early initiation of HIV treatment and given the choice to start ART irrespective of their CD4 count. Enrolment in the pilot study started in early 2014, and the study aims to enrol 300 PWID, with follow-up for 12 months. Consultation and interviews with PWID and health care workers before and during the follow-up are expected to inform future policy development.

## Transforming Service Delivery Approaches

### Integrating HIV Services into Primary Health Care

In 2010, UNAIDS/WHO proposed Treatment 2.0 to catalyse further scale-up of HIV treatment, promoting innovation in drugs and diagnostics and decentralizing HIV services closer to patients and their communities [37]. Vietnam started a pilot of Treatment 2.0 in 2012, with the goal of earlier access to HIV testing and treatment and integration of HIV services into primary health care systems (Table 1) [38, 39]. The pilot demonstrated that HIV services can be effectively delivered in commune health stations (primary health care facilities at sub-district level). While HIV testing had previously been offered only at provincial and district facilities, the pilot showed that commune health staff, with adequate training and supervision, can accurately confirm HIV testing results using the algorithm based on three rapid tests. Patients diagnosed with HIV initiated ART at district facilities and received monthly follow-up and ARV drugs at commune health station, with visits to district facilities every 6 months. In the first 9 months of the pilot, 3820 people received HIV testing, counselling and results, consisting of 1201 people from key populations including their sexual partners and 2619 pregnant women. In this pilot, people diagnosed at commune health stations and linked to care had significantly higher median CD4 counts at ART initiation than those diagnosed at district facilities (294 vs 88 cells/mm^3^) [2, 38]. Introduction of point-of-care CD4 count technology shortened the duration from CD4 testing to provision of results; for example, in Tuan Giao district, Dien Bien province, it was reduced from a median of 109 days to the same day notification [38]. VAAC is now expanding the model to other provinces. The Treatment 2.0 pilot provides important lessons learned on how Vietnam can promote earlier access to ART, and enhance sustainability through integrating HIV services into the primary health care system.

### ‘Test-and-Treat’ in Remote Mountainous Provinces

Vietnam also plans to implement an additional service delivery model to promote early access to HIV testing and ART; that is, a test-and-treat approach with immediate ART irrespective of CD4 count, for people living in remote and mountainous provinces (Table 1). In those provinces, access to CD4 testing is limited due to transportation infrastructure and location of service delivery facilities, leading to long turn-around times in some provinces and districts [38]. Combined with expanded use of rapid tests for confirmatory HIV testing at district facilities (instead of provincial facilities), the pilot aims to facilitate early uptake of HIV testing and ART and retain people across the care cascade in those hard-to-reach remote areas.

## Costs, Financing and Sustainability

The costs of ART are evolving year after year, as ARV prices decline and the number of patients increases. A costing study in Vietnam reported that costs of delivering first line ART per person-year were US$316 in the first year and US$303 in the following years [40]. The study also found that costs of ART in the first year can be less if patients start ART with CD4 count over 100 cells/mm^3^, compared with CD4 less than 100 cells/mm^3^ [40], and if ART is delivered through integrated facilities where HIV services are delivered along with other health services, compared to stand-alone facilities, which delivers only HIV services [40]. These results indicate that earlier treatment initiation and transforming service delivery could potentially improve the efficiency of ART delivery. At the same time, financing ART is critical to sustain and expand the impact of ART. Currently, more than 90 % of ARV drugs are financed by external donors, i.e. President’s Emergency Plan for AIDS Relief (PEPFAR) and Global Fund to fight AIDS, Tuberculosis and Malaria. However, external funds have started to decline since Vietnam gained middle income country status in 2010. VAAC has been working with political leaders to assure sufficient funds for the national HIV response. The Prime Minister’s Decision on sustainable financing for the HIV response [41], signed in 2013, highlighted the importance of diversifying financing sources, through mobilizing Government budget at national and provincial levels, expanding social health insurance and promoting social marketing approaches for sales of commodities and services.

## Discussion

Vietnam has a concentrated epidemic, with injecting drug use being the predominant route of HIV transmission. Vietnam has a robust national HIV response, promoting evidence-based harm reduction and ART. With growing evidence showing that ART could serve as powerful intervention to reduce HIV transmission, Vietnam has initiated attempts to harness the preventive benefits of ART. Vietnam has identified the following strategic priorities.

1. ART as part of combination prevention
  Viral load is a major determinant of HIV transmission, and available data from small studies indicate good viral suppression among people on ART in Vietnam. If Vietnam is successful in achieving high level of viral suppression among the majority of people receiving treatment, ART will likely have considerable impact on HIV transmission, as projected by the mathematical models, in addition to its proven effects to keep people healthy and alive. Financing for ART should be seen as a critical investment, which could lead to substantial reduction in HIV disease burden and relative cost-saving in the future. At the same time, focusing solely on ART may not be maximally effective, and ART should be delivered in combination with other HIV preventive services. To reach key populations and encourage them to demand earlier access to testing and ART, peer educators involved in needle syringe programmes and condom promotion play critical roles. The government of Vietnam has also committed to expanding MMT services to 80,000 PWID by 2015 [2], which will facilitate periodic HIV testing and counselling, linkages to care and ART adherence support for PWID at MMT sites. Thus, combining different prevention interventions likely enhances synergy and promote effectiveness of the programme. While analysis and debate is ongoing in Vietnam on how to best allocate limited available resources to various interventions, early access to HIV testing and ART should be considered as essential elements as part of a combination prevention approach.
2. Stay focused on key populations
  The critical question for control of the HIV epidemic is among new HIV infections, who is being infected, how, by whom and where. In Vietnam, the risk of being infected and transmitting HIV is highest in PWID, MSM, FSW and their sexual partners. The population impact of ART in preventing the transmission of HIV is substantial if these key populations are prioritized for periodic, confidential and voluntary HIV testing, provided early access to ART and supported for durable viral suppression. This does not mean that different approaches should be employed in providing ART among different populations, as all PLHIV regardless of risk status should have equal access to ART as life-saving treatment. In contrast, we propose different approaches to HIV testing for different populations. For example, periodic testing should be encouraged for key populations at least annually, as recommended by WHO [42], but not for general populations in a concentrated epidemic. Approaches for HIV testing and counselling should also be diversified, including community-based testing and counselling among key populations and provider-initiated HIV testing and counselling at MMT services, while client-initiated approaches could be used for low-risk populations [34, 43]. Expanded use of rapid HIV tests will facilitate HIV testing in community and primary health care settings and will likely increase the uptake of HIV testing, especially among key populations, who may not access HIV testing at higher level facilities. For increased testing to have an impact, though, the continuum of care for key populations also needs to be strengthened, including effective linkages of HIV-positive patients to care, timely ART initiation and long-term retention and viral suppression.
3. Enabling environment to facilitate earlier access
  There are various barriers that limit access to HIV services. PLHIV and key populations report high levels of stigma and discrimination in communities and at health facilities [3, 32, 44]. Drug users in Vietnam are also subject to compulsory drug treatment [45] and face challenges in being re-integrated into communities and finding employment. The government has made initial steps to change the policies on treatment of drug users, including development of court procedures for compulsory drug treatment at so-called 06 centers, and transition towards community-based voluntary drug-dependence treatment. Communities may also make a difference in preventing HIV transmission by developing an environment where key populations feel comfortable and can safely access HIV services. If communities use punitive measures or a discriminatory approach or provide limited support to key populations, those populations most at risk may not access HIV testing and treatment until they reach a late stage of disease. Authorities, health care workers, community-based organizations and patient groups would play important roles in transforming the communities to be more supportive and to address stigma and discrimination. They could also help key populations to gain better knowledge on treatment and create their demand for periodic HIV testing. Such concerted efforts are expected to develop enabling environment and for more PLHIV to access testing and treatment earlier and to enhance the benefits of ART.
4. Sustained political commitment
  For Vietnam to sustain and strengthen the robust HIV response, political leadership is essential. To date, the HIV response has been largely financed by external donors, and donor funding is now declining. Political commitment to control the diseases, including HIV, could drive the effective and efficient response, and could realize diversification of financing sources—tax-based financing, social health insurance and social marketing approaches—to support high-impact interventions. Vietnam’s leadership has already shown political commitment, introducing harm reduction in the national strategy (2004) and national AIDS law (2006), expanding MMT and approving the Prime Minister’s Decision to promote alternative sustainable financing for the national HIV response (2013) [41]. With political support from national and local governments and communities, Vietnam has an important opportunity to effectively control its epidemic through combination prevention directed at key populations, including targeted HIV testing and good virologic control for those on ART and through establishment of an enabling environment to support key populations’ timely access to the HIV and health services.

## Conclusion

With its demonstrated success in expanding evidence-based interventions, Vietnam aims to further strengthen its HIV response by capitalizing on preventive benefits of ART. Despite various challenges, Vietnam has the opportunity to remarkably reduce HIV transmission, especially if earlier diagnosis and treatment initiation could be achieved in key populations, in the framework of combination prevention. Political support is critically important to mobilize resources and to enhance an enabling environment for key populations.
